# Kinetics and Thermodynamics of Mg-Al Disorder in MgAl_2_O_4_-Spinel: A Review

**DOI:** 10.3390/molecules24091704

**Published:** 2019-05-01

**Authors:** Yunlu Ma, Xi Liu

**Affiliations:** 1 School of Earth and Space Sciences, Peking University, Beijing 100871, China; Yunlu.Ma@pku.edu.cn; 2 Key Laboratory of Orogenic Belts and Crustal Evolution, Ministry of Education of China, Beijing 100871, China

**Keywords:** MgAl_2_O_4_-spinel, cation exchange reaction, kinetics, temperature, thermodynamic model, non-configurational entropy

## Abstract

The MgAl_2_O_4_-spinel has wide applications in various industries and in geosciences. It shows a significant inter-site Mg-Al cation exchange (denoted by the inversion parameter *x*), which modifies structural features, such as the unit-cell parameters and the sizes of the component polyhedra, and influences the physical and chemical properties. Previous studies mainly focused on the kinetics and thermodynamics of the Mg-Al exchange reaction, with the aim to ascertain the correlation between the inversion parameter and temperature; these studies, however, reached conflicting results. Here, we first reviewed the kinetics studies on the Mg-Al cation exchange reaction, and then reviewed all thermodynamic experiments, with special attention paid to the Mg-Al cation exchange equilibrium and the quench process, which might have modified the cation distributions once attained at high temperatures. We also assessed the accuracies in the temperature measurements and in the quantifications of the *x* by different analytical methods. With some necessary temperature correction and data removal, we have landed with a generally reliable *x*-*T* dataset covering the *T*-*x* space of 873 < *T* < 1887 K and 0.18(1) < *x* < 0.357(60) (71 data pairs in total). Fitting these *x*-*T* data to three most commonly used thermodynamic models, we have obtained more accurate model parameters. Further, we also evaluated the constituent items of the Gibbs free energy for the Mg-Al cation exchange reaction with experimental results from different research fields and reached the conclusion that highly possibly the $T\Delta S_{D}$ should not be neglected. Based on this review, we suggest that: (1) Further kinetics study on the Mg-Al exchange reaction should be performed at both low *T* (<~973 K) and high *T* (>~1173 K); (2) further Mg-Al exchange equilibrium studies should be carried out at relatively low *T* and ambient *P*, as well as in vast ranges of simultaneous high *P* and high *T*; and (3) direct experimental measures about the entropies or the enthalpies of the MgAl_2_O_4_-spinels disordered to different extents should be conducted with full characterization of the starting materials and detailed description of the experimental procedures.

## 1. Introduction

The MgAl_2_O_4_-spinel has small thermal expansivity, a high melting point, excellent chemical stability, good mechanical strength, and other unique properties [1,2]. Therefore, it is widely applied as refractories [3,4], ceramic materials [5], catalysts [6,7], and humidity sensors [8] in various industries. For one example, as catalysts or catalysts support, the MgAl_2_O_4_-spinel can be used in petroleum processing and SO*_x_* emission controlling [9,10]. For another example, MgAl_2_O_4_-spinel ceramic sensors show a high sensitivity and good repeatability in highly humid environment and can therefore measure and monitor the humidity of proton exchange membrane fuel cells [11]. In the field of Earth Sciences, the MgAl_2_O_4_-spinel is a significant rock-forming mineral and an important component in the Earth’s crust and shallow mantle. Its physical, thermal, and other properties should to some extent influence the properties of the top upper mantle [12]. Its compositional features can provide important petrogenetic indications, playing important roles in some thermometrics and barometries and so on [13,14,15,16]. Further, as one of the most common end-members of natural spinel solid solutions, the MgAl_2_O_4_-spinel forms the basis for the studies of other complex spinel solid solutions. Moreover, it provides a structural model for a large number of spinel-structured minerals stable at pressure/temperature conditions of the Earth’s mantle. A noble example of these minerals with the spinel structure is ringwoodite, the dominant mineral of the lower part of the mantle transition zone (~520–660 km; [17,18,19,20]).

**Figure 1:**
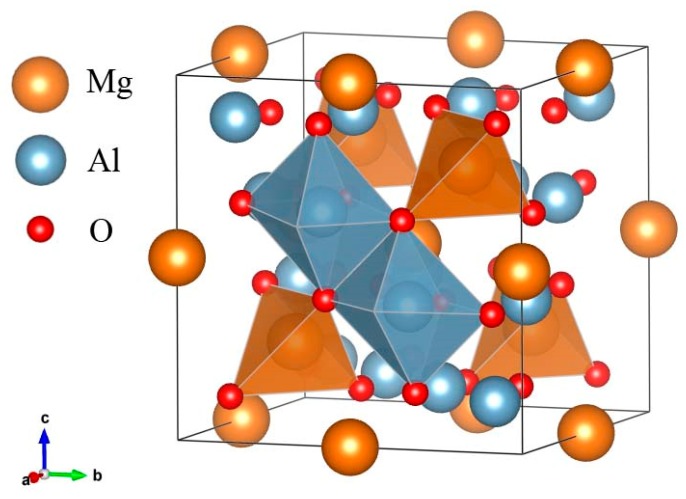
Crystallographic illustration of the MgAl_2_O_4_-spinel (normal spinel structure).

The MgAl_2_O_4_-spinel owns the classic spinel structure, which is usually assigned to the cubic space group *Fd*$\bar{3}$*m*. It is formed of an approximately cubic, close-packed array of O atoms, between which are tetrahedral T-sites and octahedral M-sites (Figure 1). The unit cell of the MgAl_2_O_4_-spinel contains 32 O atoms (with coordinates of *u*,*u*,*u* when the origin is fixed at $\bar{3}$*m*), 8 T-sites (with coordinates of 1/8,1/8,1/8) and 16 M-sites (with coordinates of 1/2,1/2,1/2). Normally, it is believed that under ambient *P*-*T* conditions, the Mg^2+^ in the MgAl_2_O_4_-spinel only occupies the T-sites, the Al^3+^ only occupies the M-sites, and the MgAl_2_O_4_-spinel is in a normal spinel configuration. However, many studies have proven that most natural MgAl_2_O_4_-spinels have inter-site cation exchange to some degrees [21,22,23,24,25,26], as described by the following reaction:

^T^Al + ^M^Mg ⇌ ^T^Mg + ^M^Al. (1)

In this situation, both Mg^2+^ and Al^3+^ appear on the T-sites and M-sites. This cation exchange is non-convergent, meaning that the above order-disorder reaction causes no change in the symmetry [27]. Therefore, the MgAl_2_O_4_-spinel can be better described by the following structural formula:

^[4]^(Mg_1−*x*_Al*_x_*)^[6]^(Mg*_x_*Al_2−*x*_)O_4_, (2)

where *x* is the degree of inversion or inversion parameter, ^[4]^ denotes the T-sites, and ^[6]^ indicates the M-sites. *x* = 0 refers to a normal spinel configuration, *x* = 2/3 corresponds to a complete disorder state, and *x* = 1 points to an inverse spinel structure. When more smaller Al^3+^ cations fill the T-sites and more larger Mg^2+^ fill the M-sites, the *x* value of the MgAl_2_O_4_-spinel increases, the mean atomic numbers of the T- and the M-sites, respectively, increase and decrease [28,29], the bond lengths of the T-O (*d*_T-O_) and M-O (*d*_M-O_), respectively, decline and extend [29,30], and the unit-cell parameter *a*, the oxygen coordinate parameter *u*, and the unit-cell volume *V* should all be reduced [29]. These structural changes should lead to some modifications of the physical properties, thermodynamic properties, and many other properties of the MgAl_2_O_4_-spinel at the macro level, such as the thermal expansion, the electrical conductivity, the vibrational spectra, and the elasticity, etc. [30,31,32,33]. Therefore, the Mg-Al cation exchange and its associating variations of the structure, physical, and thermal properties of the MgAl_2_O_4_-spinel have great value for research.

The cation order-disorder degree of the MgAl_2_O_4_-spinel, i.e., the *x* value, can be determined by various experimental techniques, including infrared spectroscopy [34], Raman spectroscopy [35,36,37,38], electron spin resonance (ESR) [39], magic-angle-spinning nuclear-magnetic resonance (MAS-NMR) [40,41], neutron powder diffraction [42,43], and single-crystal X-ray diffraction [24,29].

Existing studies on the Mg-Al disorder in the MgAl_2_O_4_-spinel mainly focused on the kinetics and thermodynamic aspects. The kinetics studies principally investigated the evolution of the *x* with time (the cation exchanging rate) and its dependence on temperature, pressure, and other variables [44,45]. The thermodynamic studies mainly aimed at ascertaining the correlations between the cation exchange reaction and the variables of temperature and pressure. Based on these correlations, the thermodynamic studies attempted establishing the thermodynamic models. Currently, experiments about the Mg-Al disorder reaction of the MgAl_2_O_4_-spinel under high pressures are still few and greatly needed [46]. In contrast, numerous studies have investigated the Mg-Al cation exchange in the MgAl_2_O_4_-spinel both under an in situ high temperature condition [24,42,43] and at ambient temperature with the quenched MgAl_2_O_4_-spinel samples once treated under high temperatures [23,29,41,47]. They reached a unanimous conclusion that the *x* value increases as the temperature is raised. However, significant discrepancies exist among these studies in terms of the temperature dependence of the *x* and the derived thermodynamic model parameters.

To address these discrepancies, this paper firstly reviewed the results from the kinetics studies. Using the conclusions from the kinetics studies, this paper secondly evaluated the results of all previous thermodynamic studies, with special attention paid to two vital aspects: (1) Whether the Mg-Al cation exchange reaction of the MgAl_2_O_4_-spinel at certain experimental temperature closely approached its thermodynamic equilibrium within the experimental heating duration; (2) whether the quench process was quick enough to fully preserve the Mg-Al cation distribution state attained by the spinel at high temperature. In addition, we also examined whether the temperature measurements were accurate or not and whether the *x* values were accurately determined by proper analytical methods or not. These exercises have eventually led to a reliable *x*-*T* dataset. Fitting these data to three most common thermodynamic models, we therefore have obtained more accurate thermodynamic model parameters for the Mg-Al cation exchange reaction of the MgAl_2_O_4_-spinel. Using these new thermodynamic models, this review further evaluated the constituent items of the Gibbs free energy and discussed whether the non-configurational entropy of the Mg-Al disordered MgAl_2_O_4_-spinel could be neglected. Finally, the pressure effect and composition influence on the *x* value, as well as the potential geological implications of the Mg-Al order-disorder exchange reaction, were briefly discussed.

## 2. Evaluation of Kinetics and Thermodynamic Studies

### 2.1. Kinetics Studies

Two kinetics studies on the Mg-Al cation exchange reaction of the MgAl_2_O_4_-spinel (Equation (1)) have been performed at high temperature and ambient pressure [44,45].

Andreozzi and Princivalle [45] defined the reaction rates of the forward and backward reactions of Equation (1) as *K*_1_ and *K*_2_ (min^−1^), respectively. In other words, their *K*_1_ referred to the reaction rate of the Al^3+^ cations on the T-sites returning to the M-sites or the Mg^2+^ cations on the M-sites returning to the T-sites, with the MgAl_2_O_4_-spinel becoming more ordered; their *K*_2_ referred to the reacting rate of the Al^3+^ cations on the M-sites transferring to the T-sites or the Mg^2+^ cations on the T-sites relocating to the M-sites, with the MgAl_2_O_4_-spinel becoming more disordered. When a full cation exchange equilibrium is attained, *K*_1_, *K*_2_, the equilibrium constant (*K*_D_) of Equation (1), and the *x* are thus in a relationship as follows:

*K*_2_/*K*_1_ = *K*_D_ = *x*^2^/[(1 − *x*) (2 − *x*)]. (3)

Consequently, the time (∆*t*) needed for an MgAl_2_O_4_-spinel to change its inversion parameter from *x*_1_ to *x*_2_ at certain temperature can be calculated with the following equation [45]:

(4) $$\Delta t=-\frac{1}{K}\int_{x_{1}}^{x_{2}}\frac{dx}{\left[x^{2}-K_{D}\left(1-x\right)\left(2-x\right)\right]}.$$

Andreozzi and Princivalle [45] then performed three sets of experiments at 973, 1073, and 1173 K to constrain the reaction rates of Equation (1). Their starting materials were synthetic MgAl_2_O_4_-spinels (Mg:Al = 1.006(7):1.996(4)) annealed at 1273 K for 12 h and drop-quenched, with the *x* value determined as 0.272(5). In the ordering experiments at lower temperatures, the MgAl_2_O_4_-spinels (*x*_1_) were annealed for different durations (∆*t*) to attain different magnitudes of Mg-Al disorder states (*x*_2_); afterwards, they were dropped into cold water. The quenching time was short enough (< 0.5 s), and the cation disordering state (*x*_2_) at high temperatures should have been well preserved. The *x* value of the quenched MgAl_2_O_4_-spinel was subsequently determined by its correlation with the oxygen parameter *u* (*x* = 21.396 − 80.714*u*; Andreozzi et al. [29]), since it is the *u* parameter that can be accurately constrained by the single-crystal X-ray diffraction method. By fitting the isothermal ∆*t*-*x* data into Equation (4), Andreozzi and Princivalle [45] calculated the *K*_1_ at different temperatures and obtained *K*_1-973_ = 0.0171(45) min^−1^, *K*_1-1073_ = 0.112(47) min^−1^, and *K*_1-1173_ = 1.12(57) min^−1^. Consequently, the *K*_1_-*T* relation was established as:

ln*K*_1_ = −23722 × (1/*T*) + 20.189. (5)

According to Andreozzi and Princivalle [45], the equilibrium *x* values of the MgAl_2_O_4_-spinels at 973, 1073, and 1173 K are 0.208(5), 0.230(5), and 0.254(5), respectively. Using Equation (3), we have therefore calculated the *K*_D_ values at these temperatures and gained *K*_D-973_ = 0.030(2), *K*_D-1073_ = 0.039(2), and *K*_D-1173_ = 0.050(2). By applying Equation (3), we have further obtained the corresponding *K*_2_ values, *K*_2-973_ = 0.0005(1) min^−1^, *K*_2-1073_ = 0.0043(18) min^−1^, and *K*_2-1173_ = 0.0555(284) min^−1^. The *K*_2_-*T* relation can then be described as:

ln*K*_2_ = −25644 × (1/*T*) + 18.744. (6)

The above analysis suggests that at 973, 1073, and 1173 K, the *K*_1_/*K*_2_ ratios are, respectively, ~33(12), 26(15), and 20(15). That is to say, the Mg-Al ordering reacting rates (*K*_1_) are much larger than the Mg-Al disordering reacting rates (*K*_2_) by, generally, one order of magnitude.

To investigate the Mg-Al cation exchange kinetics, Kashii et al. [44] performed some ordering runs at 973 and 1073 K and disordering runs at 973, 1073, 1173, and 1273 K. Their starting MgAl_2_O_4_-spinel for the ordering runs had an initial *x* value of 0.30, and that for the disordering runs had an initial *x* value of 0.05. At the end of each run, the sample was dropped into water within ~0.3 s, which could have well preserved the cation distribution state achieved at high temperatures. The ^27^Al MAS-NMR method was used to determine the *x* values of the samples by measuring the intensities of the AlO_6_ octahedra (A[6]l) and the AlO_4_ tetrahedra (A[4]l) (*x* = 2/[1 + (A[6]l/A[4]l)]). The experimental time (*t*) − A[4]l data were fitted with the least-squares method to the following equation:

(7) [A[4]l]=[A[4]lequil](1−exp[−K3(t+t0)]).

In this way, they derived the equilibrium AlO_4_ tetrahedron intensity (A[4]lequil) and reaction rate constant at certain *T*. Note that here we have denoted the reaction rate constants as *K*_3_ and *K*_4_ (*K*_3_ referring to the ordering reaction rate and *K*_4_ referring to the disordering reaction rate) for the purpose of distinguishing them from the above-defined *K*_1_ and *K*_2_. The final results (Table II in Kashii et al. [44]) indicated that the *K*_3_ values were much larger than the *K*_4_ values, by almost two orders of magnitude. In contrast, Andreozzi and Princivalle [45] suggested that the ordering rate *K*_1_ at similar temperatures was larger than the disordering rate *K*_2_ by one order of magnitude only. Further, the A[4]lequil at certain *T* derived by fitting the ordering reaction data *t*-A[4]l of Kashii et al. [44] was different from the one derived by fitting the disordering reaction data *t*-A[4]l (respectively, 0.090 and 0.079 at 973 K, and 0.104 and 0.100 at 1073 K). This might imply that the experiment results of Kashii et al. [44] had relatively larger errors.

**Figure 2:**
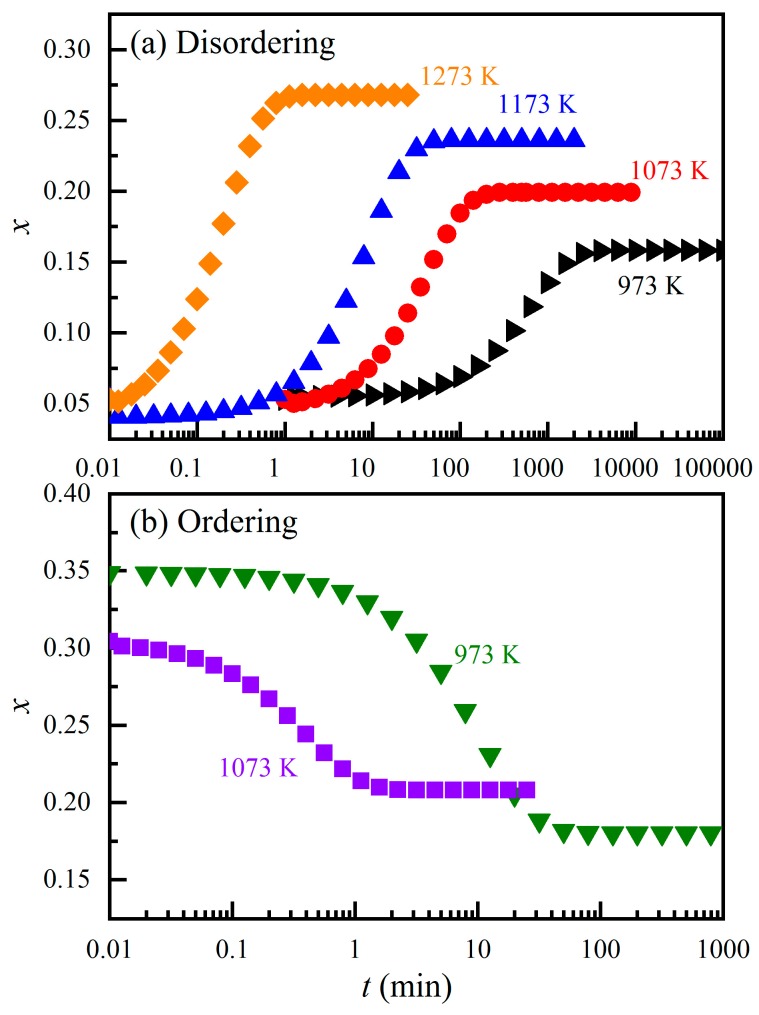
*x* versus *t* as extracted from Kashii et al. ([44]; their Table II and Equation (1)): (**a**), Mg-Al disordering reaction at different *T*; (**b**) Mg-Al ordering reaction at different *T*.

**Figure 3:**
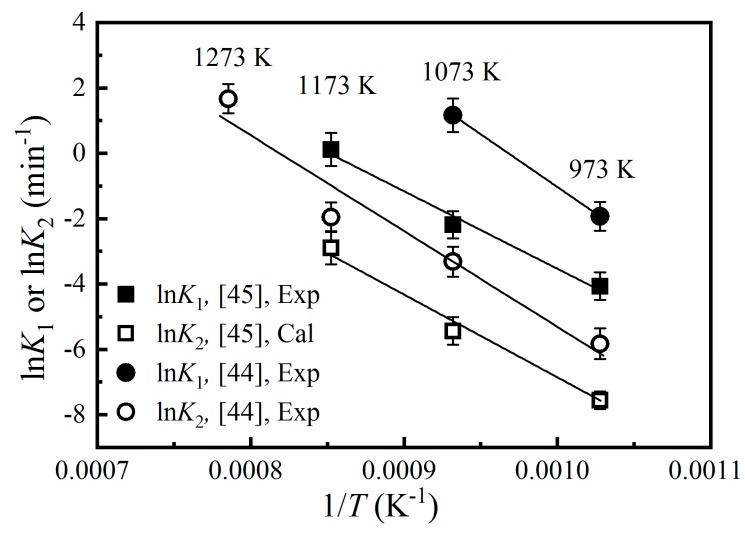
Correlations between reaction rate *K*_1_ or *K*_2_ and temperature (*T*). Both studies [44,45] suggested that *K*_1_ is much larger than *K*_2_.

To make a better comparison, we have substituted the fitting results of Kashii et al. ([44]; their Table II) back to Equation (7) and obtained the *x*-*t* data for both the cation ordering and disordering runs (Figure 2). We then fitted the *x*-*t* data to Equation (4) to derive the *K*_1_ and *K*_2_ values at relevant temperatures. The derived values were *K*_2-973_ = 0.003(1) min^−1^, *K*_2-1073_ = 0.036(17) min^−1^, *K*_2-1173_ = 0.142(64) min^−1^, *K*_2-1273_ = 5.30(237) min^−1^, *K*_1-973_ = 0.145(64) min^−1^, and *K*_1-1073_ = 3.20(163) min^−1^. Therefore, the temperature dependences of the *K*_1_ and *K*_2_ dictated by the experimental data of Kashii et al. [44] are

ln*K*_1_ = −32278 × (1/*T*) + 31.245 (8)

and

ln*K*_2_ = −29313 × (1/*T*) + 24.003, (9)

respectively. These results are apparently different to those from Andreozzi and Princivalle [45] (Figure 3). Furthermore, following Equation (3), we can use the *K*_1_ and *K*_2_ calculated with Equations (8) and (9), respectively, to obtain the *K*_D_ and *x* at a certain temperature. The results have shown that the calculated *K*_D_ and *x* both show negative relationships with temperature (in the 973–1273 K interval), which is contradictory with the well-established positive correlation between the *x* and *T* [23,24,29,40,41,42,43,45,47,48]. It follows that the ordering and disordering kinetics experiments of Kashii et al. [44] led to abnormal results. This phenomenon probably might have rooted in the analytical method (^27^Al MAS-NMR) used to determine the *x* value. Compared with the single-crystal X-ray diffraction method (uncertainty usually < 0.005), the precision of the ^27^Al MAS-NMR method to determine the *x* value is relatively lower (uncertainty up to ~0.02 according to Maekawa et al. [23]).

In the following discussion of this review, we have then chosen to use the kinetics results of Andreozzi and Princivalle [45] to evaluate the Mg-Al cation exchange thermodynamic experiments. It should be noted that the Mg-Al cation exchange reaction in the MgAl_2_O_4_-spinel is extremely slow at a low temperature but extremely fast at a high temperature. Due to this reaction feature and the limited experimental techniques, the kinetics studies of the Mg-Al cation exchange reaction of the MgAl_2_O_4_-spinel are still limited within 973–1273 K at ambient pressure. No experimental data are available at both lower temperatures and higher temperatures and at ambient pressure, and all temperature ranges under high pressures. Inevitably, we need to extrapolate the kinetics results of Andreozzi and Princivalle [45] to some extent.

### 2.2. Thermodynamic Experiments

Thermodynamic studies of the Mg-Al cation exchange reaction in the MgAl_2_O_4_-spinel aim at the relationship between the *x* and temperature, pressure, and other physical-chemical quantities. There are three critical factors to consider: (1) Whether the cation exchange equilibrium is closely approached in the experiments, (2) whether the cation order-disorder state attained at the experimental conditions can be well quenched, and (3) whether the measurements of the *x* value, temperature, pressure, and other quantities are accurate enough. In this review, we use the results from the kinetics study of Andreozzi and Princivalle [45] to judge the cation exchange equilibrium state at certain temperature and pressure, and to evaluate the possible quench effect on the *x*. In addition, we also assess the accuracy in the temperature and *x* value measurements.

**Figure 4:**
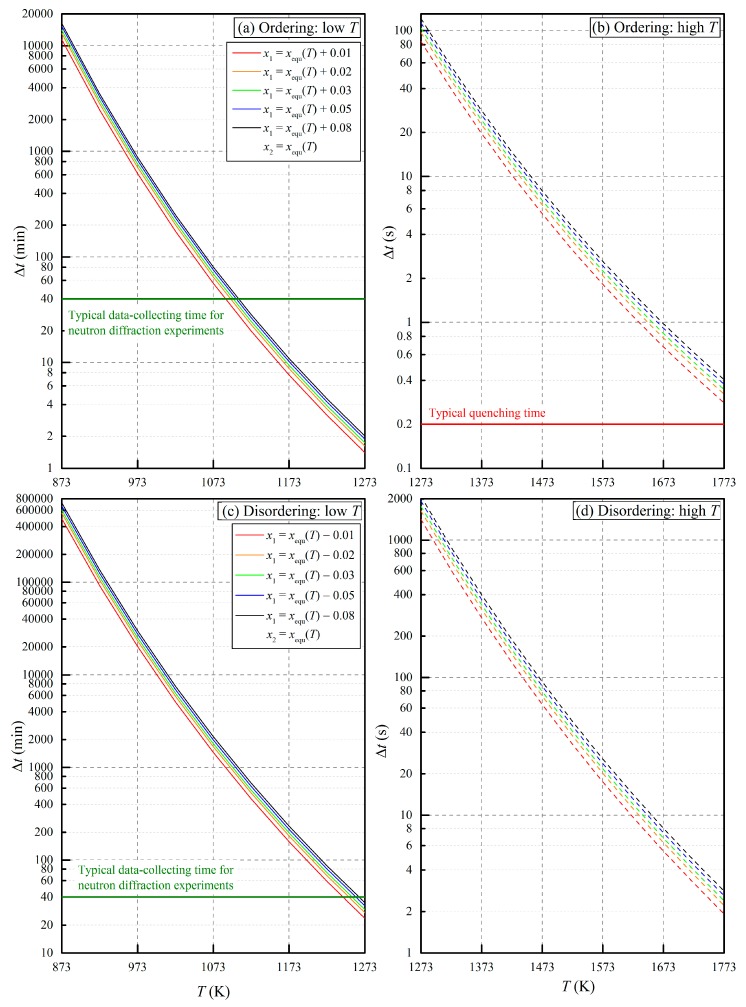
Required time (∆*t*) for the MgAl_2_O_4_-spinels with initial *x* values different to the equilibrium *x* values by 0.01, 0.02, 0.03, 0.05, and 0.08 to reach their equilibrium at different temperatures (*T*) and at room pressure. (**a**) Cation-ordering reaction in the *T* range of 873–1273 K; (**b**) cation-ordering reaction in the *T* range of 1273–1773 K; (**c**) cation-disordering reaction in the *T* range of 873–1273 K; (**d**) cation-disordering reaction in the *T* range of 1273-1773 K. All calculations were based on the kinetics study of Andreozzi and Princivalle [45], with inevitable extrapolations both to low *T* and high *T*. Due to the relatively long-distance extrapolation at high *T*, the ∆*t*-*T* curves at high temperatures are shown as dashed lines in (**b**,**d**). Note that the typical quench time in the experiments performed with a high-*T* furnace (~0.2 s), and the typical data-collecting time in the neutron diffraction experiments (~40 min) have been sketched in (**b**), and (**a**), and (**c**), respectively.

To facilitate the evaluation, we have calculated the time intervals needed for certain amounts of Mg-Al cation exchanging in a large range of temperatures (ambient pressure), starting from different initial *x* values (*x*_1_ = *x*_equ_(*T*) ± 0.01/0.02/0.03/0.05/0.08) to approach the final equilibrium *x* values (*x*_2_ = *x*_equ_(*T*)) at certain temperatures. The results are shown in Figure 4. Clearly, all the major conclusions from the kinetics study of Andreozzi and Princivalle [45] have been illustrated in this figure. By comparing Figure 4a,b, respectively, to Figure 4c,d, for example, one can tell that the Mg-Al cation ordering reaction is much faster than the disordering reaction at the same temperature. Another major feature shown in Figure 4 is the strong temperature dependence of the Mg-Al cation exchanging rate: A few minutes or even seconds of heating at temperatures >1573 K can significantly alter the *x* value, whereas several days or even months at temperatures <873 K can change the *x* value little. This implies that at high *T*, one does not need to worry too much about the experimental equilibrium state but should seriously evaluate the quench modification. At low *T*, the equilibrium state of the experiments becomes the major concern. Extra points shown by Figure 4 include: (1) If the quench process can be kept as short as less than 0.2 s (shown as red line in Figure 4b), the quench modification on the *x* value is generally negligible; (2) if the data-collecting time in the neutron diffraction experiments is close to 40 min (shown as green lines in Figure 4a,c), the cation-ordering (or cation-disordering) experiments at temperatures <~1073 (or <~1273) K might not approach their close equilibrium and the neutron diffraction data should not be trusted, the experiments at temperatures around 1073 K (or 1273 K) may vary the *x* values with time and then the neutron diffraction data record signals from a sample with a range of different and transient *x* values, but the experiments at higher temperatures such as 1173 K (or 1373 K) can quickly reach their equilibrium and the neutron diffraction data can generally reflect the equilibrium *x* at those temperatures.

#### 2.2.1. Mg-Al Cation Exchange Equilibrium and Quench Effects

Wood et al. [47] studied the ordering and disordering processes of the Mg-Al cation exchange reaction in the temperature range of 983–1473 K and at ambient pressure. Their MgAl_2_O_4_-spinels equilibrated at high temperatures were quenched by dropped into water, and the *x* values were determined by means of the ^27^Al MAS-NMR method. Their starting MgAl_2_O_4_-spinels were synthesized at 1573 K and 1 atm, at 1698 K and 1 atm, and at 937 K and 1 kbar, with the *x* values determined as 0.37(4), 0.39(4), and 0.21(2), respectively. The cation exchange equilibrating experiments were performed at 983–1473 K for 2~400 h. Among these experiments, two cation-ordering ones using the MgAl_2_O_4_-spinels of *x* = 0.39(4) as the starting materials were conducted at 983 K and run for 2 and 15 h. The final *x* values were, respectively, 0.28(3) and 0.26(3). According to Figure 4a, ~700 min is needed for an MgAl_2_O_4_-spinel with an initial *x* value higher than the equilibrium *x* value by 0.08 to reach its cation exchange equilibrium at 983 K. We therefore tend to believe that these two runs did not closely reach their cation exchange equilibrium. In contrast, we believe that the two ordering and disordering runs at 988 K both for 370 h, with the starting *x* values of, respectively, 0.39(4) and 0.21(2) and the final *x* values of, respectively, 0.26(3) and 0.24(2), reached their cation exchange equilibrium (Figure 4a,c). In a similar way, we think that the runs at 1073, 1123, 1173, 1323, and 1473 K all attained close cation exchange equilibrium. At the end of these experiments, the MgAl_2_O_4_-spinels were quenched within ~2 s by dropped into water, and the high-*T* cation order-disorder states should not be significantly modified by the quench process (Figure 4). Indeed, the differences of the *x* values demonstrated by the ordering and disordering runs at 988, 1073, 1123, and 1323 K were, respectively, 0.02, 0, 0, and 0.03 (data precision was ±10%, i.e., <0.039), suggesting good cation exchange equilibrium and effective quench process in these experiments.

Peterson et al. [42] studied the Mg-Al cation exchange reaction in the MgAl_2_O_4_-spinel at 873–1273 K and at ambient pressure. Their MgAl_2_O_4_-spinels were equilibrated at high temperatures and characterized by in situ neutron powder diffraction method. The initial *x* value of their sample W was 0.32(2). Their experiments were conducted from high temperature to low temperature. However, the specific temperature decreasing rate, waiting time before data collection, and neutron diffraction data-collecting time at every experimental temperature were not reported. If we take as references those data-collecting parameters from Redfern et al. [43], in which the neutron powder diffraction method was also used, we may assume that the data-collecting time in Peterson et al. [42] was ~40 min. According to Figure 4a,c, we then reach the conclusion that the runs at 1273 and 1173 K were in good cation exchange equilibrium (the final *x* values were, respectively, 0.36(1) and 0.33(2)) while the runs at 1073, 973, and 873 K did not closely reach their cation exchange equilibrium.

Millard et al. [41] investigated the temperature dependence of the *x* in the MgAl_2_O_4_-spinel in the 973–1675 K interval and at room pressure. Their MgAl_2_O_4_-spinels were equilibrated at high temperatures and quenched in liquid N_2_, with the *x* values determined by the ^27^Al and ^17^O MAS-NMR methods. Their starting MgAl_2_O_4_-spinel was synthesized at ~1573 K (*x* = 0.26(3)). They performed both Mg-Al cation ordering experiments and disordering experiments, with the former experiments at 973, 1073, 1080, and 1175 K for 180–528 h and the latter experiments at ~1273, ~1473, and ~1673 K for 29–471 h. According to Figure 4a,c, we believe that these experiments had reached good cation exchange equilibrium. At the end of these experiments, the MgAl_2_O_4_-spinels equilibrated at 973, 1073, 1080, and 1175 K were removed and quenched in liquid N_2_ within 5–10 s, and the MgAl_2_O_4_-spinels heated at ~1273, ~1473, and ~1673 K were directly quenched by dropped into liquid N_2_ within ~2 s. The kinetics results indicate that the MgAl_2_O_4_-spinel at ~1573 K, with an initial *x* value higher than the equilibrium *x* value by 0.01, should approach its cation exchange equilibrium within ~1.7 s (Figure 4b). That is to say, the MgAl_2_O_4_-spinel treated at ~1673 K and quenched within 2 s might have altered its *x* value during the quench process. The 2 s-quench process of other runs at 973–1473 K should not significantly change the *x* values. Indeed, the differences of the *x* values of the MgAl_2_O_4_-spinels from the ordering and disordering runs at 1173, ~1273, and ~1473 K were, respectively, 0.02, 0, and 0.02 (with a data error of ±0.03), suggesting that these experiments reached good cation exchange equilibrium and were quenched well.

Maekawa et al. [23] conducted some experiments on the Mg-Al cation exchange process in the MgAl_2_O_4_-spinel at room pressure and in the 973–1887 K interval. The initial *x* value of their starting materials was 0.050(15). On one hand, Maekawa et al. [23] annealed their MgAl_2_O_4_-spinels at 973–1369 K and quenched them within ~0.2 s. These experiments were later reported in Kashii et al. [44]. As previously analyzed in this review, they led to negative relationships between the temperature and *K*_D_ as well as *x*, which are contradictory with the conclusions from all other studies. We therefore suspect the reliability of the results from these experiments. On the other hand, Maekawa et al. [23] ground the MgAl_2_O_4_-spinel into a powder, heated it at 1484–1887 K, and in situ determined the *x* values at high temperatures using the ^27^Al MAS-NMR method. However, they did not report the specific experimental details such as the experimentation *T*-sequence (i.e., from high to low temperature or from low to high temperature), the heating time before the spectrum-collecting, and the spectrum-collecting time of the ^27^Al MAS-NMR method, and so on. According to Figure 4b,d, the MgAl_2_O_4_-spinel, with an initial *x* value higher and lower than the equilibrium *x* value by 0.08, should close approach its cation exchange equilibrium at 1484 K within ~8 and ~80 s, respectively. If we assume that collecting one ^27^Al MAS-NMR spectrum might cost ~60–600 s (private communication with Professor Wei Li from Nanjing University), the experiments with a heating sequence from high to low temperature might have reached their cation exchange equilibrium whereas those with a heating sequence from low to high temperature might have not. Anyhow, the high-temperature ^27^Al MAS-NMR spectra showed some additional narrow peaks and contained some obvious noises. To analyze these spectra further, the line shape-simulating parameters used for ambient conditions had to be extrapolated to high temperatures. Eventually, these high temperature spectra resulted in somewhat large uncertainties in the *x* values (up to ±0.06), so that the close cation exchange equilibrium in the experiments was not important any more. Nevertheless, we have chosen to trust this set of high temperature data from Maekawa et al. [23], considering the rarity of the *x*-*T* data at very high temperatures (e.g., 1484–1887 K) for the cation exchange reaction of the MgAl_2_O_4_-spinel.

Redfern et al. [43] investigated the Mg-Al cation exchange reaction in the MgAl_2_O_4_-spinel at the temperature range of 364–1873 K and at ambient pressure. They used the neutron powder diffraction method to in situ determine the *x* values of their MgAl_2_O_4_-spinels. Redfern et al. [43] prepared two batches of starting MgAl_2_O_4_-spinels, sample S (MgAl_2_O_4_ and *x* = 0.218(8)) and sample N (Mg_0.99_Al_2_O_4_ and *x* = 0.199(9)). They studied the Mg-Al cation ordering and disordering processes with increasing and decreasing experimental temperatures (with a *T*-changing ramp of ~1 K·min^−1^), respectively. The collecting time for one neutron diffraction spectrum was ~40 min, but the waiting time before the data-collecting was not reported. According to Figure 4, the runs at *T* > 1273 K in the heating-up experimental sequence and those at *T* > 1123 K in the cooling-down experimental sequence should all have reached good cation exchange equilibrium. The differences of the *x* values obtained from the ordering and disordering experiments at similar temperatures > 1152 K were < 0.035 (data precision < 0.024), indicating good cation exchange equilibrium states established in these experiments indeed.

Andreozzi et al. [29] took one batch of synthetic MgAl_2_O_4_-spinels (*x* = 0.229(6)) as the starting material to conduct some disordering experiments at 1073–1373 K and at ambient pressure on one hand. The heating durations were all 1 d. On the other hand, they used another batch of synthetic MgAl_2_O_4_-spinels (*x* = 0.29(1)) to conduct some ordering experiments at room pressure and at 1223, 1073, 973, and 873 K, with the experimental times being 3, 7, 90, and 45 d, respectively. According to Figure 4, all these experiments should have reached good cation exchange equilibrium. When these runs ended, the MgAl_2_O_4_-spinel samples were dropped into cold water and quenched within less than 0.5 s (from 1373 to 673 K). The kinetics results suggest that this quench process should have well preserved the cation distributions attained at high temperatures (Figure 4). Andreozzi et al. [29] accurately determined the *u* values of their MgAl_2_O_4_-spinels using the single-crystal X-ray diffraction method and converted these values into the *x* values by means of the bond length method [21]. The differences of the *x* values acquired from the ordering and disordering runs at 1087 and 1223 K were, respectively, 0.001 and 0.004 (data precision < 0.01), suggesting that these runs all reached close cation exchange equilibrium and were quenched well.

Carbonin et al. [24] studied the Mg-Al cation exchange reaction in the MgAl_2_O_4_-spinel in the 473–1223 K interval at room pressure. They used the single-crystal X-ray diffraction method to in situ determine the *u* values of the MgAl_2_O_4_-spinels first and then derived the *x* values later based on the bond length method [21]. One of their staring materials was a synthetic MgAl_2_O_4_-spinel (SYN; *x* = 0.243). With this spinel, Carbonin et al. [24] conducted both heating-up and cooling-down experiment sequences. For the runs at 973 K, the MgAl_2_O_4_-spinel was heated in the hearing-up and cooling-down experimental sequences for 14.5 h and 16 h, respectively, before the X-ray data was collected. For the run at 1073 K in the cooling-down experimental sequence, the waiting time was ~4.3 h before the X-ray data was collected. In all other runs at other temperatures, the MgAl_2_O_4_-spinel was heated for ~2–2.4 h and then characterized. Figure 4 suggests that the runs in the *T* range of 1073–1223 K should have reached good cation exchange equilibrium. The small differences of the *x* values demonstrated by the heating-up and cooling-down runs at identical temperatures also implied good cation exchange equilibrium (0.011 and 0.006 at 1073 and 1173 K, respectively; data precision ≤ 0.01 according to Andreozzi et al. [29]).

#### 2.2.2. Temperature Measurements and Determination of x Value

**Figure 5:**
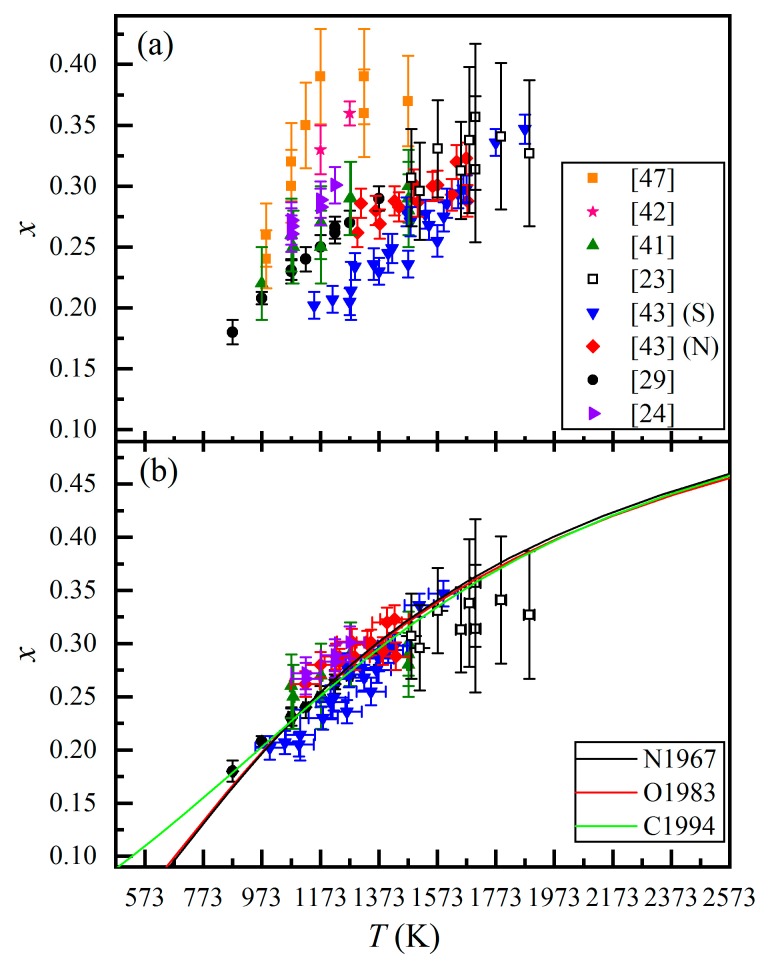
(**a**) Correlations between temperature (*T*) and *x* of the MgAl_2_O_4_-spinel from equilibrium experiments, in which the cation exchange reaction closely reached its equilibrium and the cation order-disorder state was perfectly preserved by the quenching process. (**b**) Correlations between temperature (*T*) and *x* of the MgAl_2_O_4_-spinel from equilibrium experiments, with further temperature correction, data comparison, and selection (see text for the details). Two samples, sample S and sample N, were investigated by Redfern et al. [43]. N1967 = the Navrotsky and Kleppa thermodynamic model [49], O1983 = the O’Neill and Navrotsky thermodynamic model [50], C1994 = the Carpenter and Salje thermodynamic model [51].

With the above critical evaluation of the cation exchange equilibrium data on the basis of the kinetics results, we have obtained 84 pairs of *x*-*T* data for the Mg-Al cation exchange equilibrium in the MgAl_2_O_4_-spinel at ambient pressure (see Table S1 for the details). These data are summarized in Figure 5a. As is clear in Figure 5a, these *x*-*T* data do not fall on a nice trend, suggesting that other variables such as the temperature measurement and the accuracy of the *x* values determined by different analytical methods need to be evaluated.

Accurate temperature measurement is critical. Both Wood et al. [47] and Andreozzi et al. [29] used the Pt-Rh thermocouple to measure their experimental temperatures. The Pt-Rh thermocouple is applicable for the *T* range of 323–2041 K [52], with a precision of ~±5 K in the *T* range of 873–973 K and ~±10 K for higher temperatures [29]. Millard et al. [41] did not mention their thermocouple type but stated that a muffle furnace was used to anneal their MgAl_2_O_4_-spinel. In general, the temperature control of the muffle furnace is relatively accurate (±5 K). Peterson et al. [42], Maekawa et al. [23], Redfern et al. [43], and Carbonin et al. [24] all conducted in situ high temperature experiments to study the Mg-Al cation exchange reaction. Among them, Maekawa et al. [23] adopted an optical pyrometer (IR-FB) to monitor the temperature, and the monitoring accuracy was ±1%. In comparison, all other studies used thermocouples to measure temperature. Due to the certain distance between the tip of the thermocouple and the data-collecting point of the MgAl_2_O_4_-spinel, a temperature gradient was expected, and the accuracy of the temperature measuring was therefore relatively low. Carbonin et al. [24] used a special microfurnace reported by Molin et al. [53] to heat their MgAl_2_O_4_-spinel and employed a Pt-Rh thermocouple to measure their temperature. According to Molin et al. [53], the thermocouple readings in Carbonin et al. [24] might be ~100 K lower than the real experimental temperatures for the *T* range of 1073–1223 K. Accordingly, it seems appropriate to raise the measured temperatures by 50 K and set their error as ±50 K. With this correction, the *x*-*T* data of Carbonin et al. [24] are in good accordance with the data from Andreozzi et al. [29] (Figure 5b). As for Peterson et al. [42], they did not report any relevant information about their temperature measurement. However, in view of their experiment temperature range (<1273 K) and the general features of common thermocouples in this temperature range, we believe that the accuracy of their temperature measurements was mainly dependent on the distance between the tip of the thermocouple and the data-collecting point of the MgAl_2_O_4_-spinel. However, it is difficult to evaluate the effect of this factor for the lack of adequate description of the experimental cell arrangements in Peterson et al. [42], and in Redfern et al. [43] as well.

The temperature measurement in Redfern et al. [43] had another problem. It was done with the K-type thermocouple, which is applicable in the *T* interval of 3~1603 K [52]. When the temperature is higher than 1603 K, the negative leg of this type of thermocouple melts and the thermocouple cannot function properly any more. However, the highest temperature recorded by the K-type thermocouple in Redfern et al. [43] was 1873 K, a temperature significantly higher than the upper limit of the proper functioning temperature range. Consequently, we tend to believe that most temperature measurements (*T*_measure_) in Redfern et al. [43] were higher than the actual experimental temperatures (*T*_actual_) and should be corrected in some ways. At room temperature, the thermocouple reading should faithfully reflect the experimental temperature (*T*_measure_ = *T*_actual_ = 298 K). As the experimental *T* increased, the difference between the real experimental *T* and the thermocouple reading should have been increased because the thermal gradient between the tip of the thermocouple and the data-collecting point of the sample increased. To allow the K-type thermocouple to function at the highest recorded *T*, we then assume rather arbitrarily that the actual temperature in the experiment with the highest recorded *T* (1873 K) was ~8 K lower than the upper limit of the proper functioning temperatures of the K-type thermocouple (i.e., 1603 K): *T*_measure_ = 1873 K and *T*_actual_ = 1595 K, with a difference of ~278 K at the recorded experimental *T* of 1873 K. To simplify the issue, we further assume that there was a linear correlation between the recorded experimental *T* and the *T* difference and eventually reach the following *T*-correction equation:

*T*_actual_ = 0.8235 × *T*_measure_ + 52.60. (10)

In addition, we set the temperature error as ±50 K. With our correction, the *x*-*T* data of Redfern et al. [43] seems compatible with the data from Andreozzi et al. [29] (Figure 5b). As some examples, when *T*_measure_ = 1291 K and *x* = 0.234(11), and *T*_measure_ = 1662 K, and *x* = 0.298(12), the real experimental temperatures should have been ~1114 and 1419 K, respectively. For the *x* values of 0.240(10) and 0.290(10), correspondingly, the temperatures from Andreozzi et al. [29] were *T* = 1123 K and *T* = 1373 K, respectively. It follows that our *T* correction to the recorded temperatures in Redfern et al. [43] appears reasonable.

The relative accuracy in the *x* values determined using different characterizing methods in different studies is hard to gauge. When the MgAl_2_O_4_-spinel is equilibrated at certain temperature, its determined *x* values by means of different analytical methods should not be distinctly different. Wood et al. [47], Millard et al. [41], and Maekawa et al. [23] all adopted the MAS-NMR method to determine the *x* values of the MgAl_2_O_4_-spinel. However, Millard at al. [41] and Maekawa et al. [23] pointed out that Wood et al. [47] used somewhat different NMR acquisition parameters to collect their data, which led to obviously larger *x* values (Figure 5a). In addition, the *x* values at 1273 and 1173 K (0.36(1) and 0.33(2), respectively) from Peterson et al. [42] are clearly higher than those from all other studies (Figure 5a), with the specific reason unknown. As previously mentioned, there might be a problem in their temperature measurements resulted from the distance between the tip of their thermocouple and the data-collecting part of their MgAl_2_O_4_-spinel. Nevertheless, we have decided to leave these data out of our final *x*-*T* dataset.

With the above temperature corrections to the experiments in Carbonin et al. [24] and in Redfern et al. [43], and the elimination of those data from Wood et al. [47] and Peterson et al. [42], we have finally arrived at an *x*-*T* dataset (71 data pairs in total) for the Mg-Al cation exchange equilibrium of the MgAl_2_O_4_-spinel at room pressure (Figure 5b). This dataset covers the temperature range of 873–1887 K and the *x* range of 0.18(1)–0.357(60). We will use it to extract the thermodynamic model parameters which may be more accurate and reliable, as the data points form a generally good trend (Figure 5b).

### 2.3. Thermodynamic Models

The Mg-Al cation exchange reaction in the MgAl_2_O_4_-spinel should influence the thermal properties, including the Gibbs free energy (G), configurational entropy ($S_{C}$) and non-configurational entropy ($S_{D}$), internal energy (U), and enthalpy (H) and so on. Different thermodynamic models were put forward to describe the relationships between these thermal parameters of the MgAl_2_O_4_-spinel and its inversion parameter *x* [49,50,51,54,55]. The common theoretical basis of these models is the change of the Gibbs free energy caused by the Mg-Al cation exchange ($\Delta G_{D}$):

(11) $$\Delta G_{D}=\Delta U_{D}-T\left(\Delta S_{C}+\Delta S_{D}\right)+P\Delta V_{D},$$

where $\Delta U_{D}+P\Delta V_{D}=\Delta H_{D}$, and $S_{C}$ can be calculated by:

(12) $$S_{C}=-R\sum b^{s}N_{i}^{s}\;lnN_{i}^{s},$$

with the $N_{i}^{s}$ standing for the fraction of the specie i in the site s, the $b^{s}$ referring to the number of the s site per formula unit, and the R being the gas constant. If we choose the normal spinel (i.e., *x* = 0) at some certain temperature and pressure as the standard state, then the cation exchange reaction for the (Mg_1-*x*_Al*_x_*)(Mg*_x_*Al_2-*x*_)O_4_ should lead to a change in the $S_{C}$, as described by the following equation:

(13) $$\Delta S_{C}=-R\left[x\ln x+\left(1-x\right)\ln\left(1-x\right)+xln\left(\frac{x}{2}\right)+\left(2-x\right)\ln\left(1-\frac{x}{2}\right)\right].$$

Differences among the existing thermodynamic models mainly originated from whether the $\Delta S_{D}$ term in Equation (11) could be neglected or not and from the exact formula used to describe the relationships between the *x* and the $\Delta U_{D}$ and $\Delta H_{D}$. In this review, we will briefly introduce the three most common thermodynamic models and derive new parameters for them using those *x*-*T* data passing our evaluation (Figure 5b). It is worth noting that, in view of the relatively narrow range of the *x*-*T* data (0.18(1) < *x* < 0.357(60) and 873 < *T* < 1887 K) and the different accuracies in the data from different investigations, there is still room for further improvement in the thermodynamic model parameters.

#### 2.3.1. Navrotsky and Kleppa model

Navrotsky and Kleppa [49] assumed that the cation distributions in the T-sites and the M-sites were both ideal, so that the cation exchange only affected the $T\Delta S_{C}$ (i.e., the $T\Delta S_{D}$ being negligible). As a result, the $\Delta H_{D}$ in Equation (11) was only correlated with the $T\Delta S_{C}$. Next, they made the assumption that the $\Delta H_{D}$ had a linear correlation with *x*, $\Delta H_{D}=x\Delta H_{D,\mathrm{int}}$, where $\Delta H_{D,\mathrm{int}}$ was the enthalpy change from a normal spinel structure to an inverse spinel structure. When the cation exchange reaches the equilibrium at certain temperature and pressure, there should be $\partial \Delta G_{D}/\partial x=0$, and substituting Equation (13) into Equation (11) leads to

(14) $$-\frac{\Delta H_{D,\mathrm{int}}}{RT}=\ln\frac{x^{2}}{\left(1-x\right)\left(2-x\right)}.$$

Using a weighted least-squares regression method, we have fitted the *x*-*T* data shown in Figure 5b to Equation (14), and we have obtained $\Delta H_{D,\mathrm{int}}$ = 29.30(19) kJ·mol^−1^ (R^2^ = 0.850). In comparison, Millard et al. [41] reported $\Delta H_{D,\mathrm{int}}$ = 28(1) kJ·mol^−1^, and Navrotsky and Kleppa [49] obtained $\Delta H_{D,\mathrm{int}}$ = −43.55(2726) kJ·mol^−1^ using their solution calorimetry data collected from some MgAl_2_O_4_-spinels with different *x* values (more discussions in Section 3.1). These results are consistent with ours within error. 

Our new parameters for the Navrotsky and Kleppa model [49] thus suggest that when 1 mol MgAl_2_O_4_-spinel changes from a normal structure to a fully disorder structure (the *x* value varying from 0 to 2/3), the $\Delta H_{D}$ is ~19.53(13) kJ.

#### 2.3.2. O’Neill and Navrotsky model

O’Neill and Navrotsky [50] also thought that the $T\Delta S_{D}$ was usually very small and could be neglected. In addition, they pointed out that the volume change ($\Delta V_{D}$) and the corresponding energy change ($P\Delta V_{D}$) caused by the cation exchange reaction in the MgAl_2_O_4_-spinel at room pressure were very small and could be neglected. Their decomposition of the internal energy (U) into different components and subsequent analyses suggested that the $\Delta U_{D}$ was a quadratic function about the *x*, $\Delta U_{D}=\alpha x+\beta x^{2}$, with two parameters *α* and *β*. They further pointed out that the parameters *α* and *β* were generally expected to be of approximately equal magnitude and opposite sign. Thus, when the cation exchange reaction reaches its equilibrium at some certain temperature and pressure ($\partial \Delta G_{D}/\partial x=0$), Equation (11) could be expressed as:

(15) $$-RTln\left(\frac{x^{2}}{\left(1-x\right)\left(2-x\right)}\right)=\alpha +2\beta x.$$

Based on a weighted least-squares regression method, fitting our previously evaluated *x*-*T* data (Figure 5b) to Equation (15) yields α = 28.63(136) kJ·mol^−1^ and β = 1.35(261) kJ·mol^−1^ (R^2^ = 0.840). The value of the parameter β is close to 0, which to some extent justifies the $\Delta H_{D}=x\Delta H_{D,\mathrm{int}}$ assumption made by Navrotsky and Kleppa [49]. Furthermore, the value of the parameter α derived here is essentially identical to the value of the above-derived $\Delta H_{D,\mathrm{int}}$ (29.30(19) kJ·mol^−1^) for the Navrotsky and Kleppa model [49], suggesting that these two models can treat our *x*-*T* data equally well, as shown in Figure 5b.

**Figure 6:**
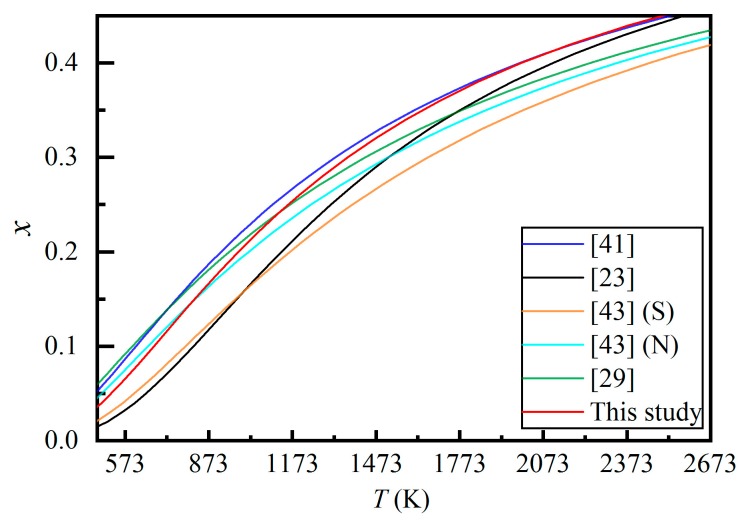
O’Neill and Navrotsky thermodynamic model [50] calibrated by different studies. Two samples, sample S and sample N, were investigated by Redfern et al. [43].

The O’Neill and Navrotsky model [50] is by far the most commonly used model in the studies of the Mg-Al cation exchange reaction of the MgAl_2_O_4_-spinel. In the past (Figure 6), Millard et al. [41] obtained α = 25(5) kJ·mol^−1^ and β = 5.8(95) kJ·mol^−1^; Maekawa et al. [23] obtained α = 35(5) kJ·mol^−1^ and β = −32(5) kJ·mol^−1^ (with their experimental data, we have, however, obtained α = 36.17(109) kJ·mol^−1^ and β = −5.96(237) kJ·mol^−1^, which are shown in the figure); Redfern et al. [43] obtained α = 32.8(9) kJ·mol^−1^ and β = 4.7(20) kJ·mol^−1^ for their sample S and α = 25.7(14) kJ·mol^−1^ and β = 11.4(28) kJ·mol^−1^ for their sample N; Andreozzi et al. [29] obtained α = 23(2) kJ·mol^−1^ and β = 13(4) kJ·mol^−1^. As shown in Figure 6, the thermodynamic model by Millard et al. [41] is very similar to ours at *T* > ~1323 K, but the difference increases as *T* decreases, reflecting the fact that no experimental data at relatively low *T* was used in deriving the α and β parameters by Millard et al. [41]. In contrast, the thermodynamic model by Andreozzi et al. [29] is very close to ours at ~1473 < *T* < 873 K, but it displays a gradually increasing difference to ours at both higher and lower *T*, indicating the importance of the data at both higher and lower *T* in deriving the α and β parameters, which were not experimentally constrained by Andreozzi et al. [29]. Moreover, the thermodynamic models by Redfern et al. [43], especially the one for the sample N, are generally identical to ours at *T* < ~900 K, but demonstrate increasing difference when *T* increases, which implies the *T* measurement by the K-type thermocouple becoming increasingly worse; if there were no such a *T*-measurement problem, their models would have been the bests because of the wide *T*-*x* data range covered by their experiments. The thermodynamics model by Maekawa et al. [23] appears in good agreement with ours at extremely high *T* such as 2500 K, illustrating the merits of their in situ experimental data at very high *T*.

#### 2.3.3. Carpentar and Salje Model

Using Landau’s theory of phase transitions [56], Carpenter and Salje [51] adopted the order parameter (Q) to describe the order-disorder degree of the cation exchange reaction in the spinel. For the MgAl_2_O_4_-spinel, the order parameter is defined as $Q=\left|X_{\mathrm{Al}}^{M}-X_{\mathrm{Al}}^{T}\right|=\left|X_{\mathrm{Mg}}^{M}-X_{\mathrm{Mg}}^{T}\right|$, where $X_{\mathrm{Al}}^{M}$ is the proportion (between 0 and 1) of the Al^3+^ on the M-sites, etc. Q = 0 refers to the fully disordered spinel structure, and Q = 1 stands for the fully ordered structure. The value of Q can be converted into the value of *x* by $x=\frac{2}{3}(1-Q$). Carpenter and Salje [51] expressed the $\Delta G_{D}$ as:

(16) $$\Delta G_{D}=-hQ+\frac{1}{2}a\left(T-T_{c}\right)Q^{2}+\frac{1}{6}cQ^{6},$$

where *a* and *c* were the standard Landau coefficients, *h* referred to the effective field, and $T_{c}$ was the critical temperature, which was related to the pairwise interaction energy between the nearest neighbors [55,57]. The physical meaning of Equation (16) corresponded to Equation (11). The term $-\frac{1}{2}aQ^{2}$ stood for the cation exchange entropy caused by the spinel cation-ordering changing from the fully disordered structure (Q = 0) to the somewhat ordered structure (Q). Furthermore, the rest $-hQ-\frac{1}{2}aT_{c}Q^{2}+\frac{1}{6}cQ^{6}$ referred to the cation exchange enthalpy ($\Delta H_{D}$; Carpenter et al. [55]). When the cation exchange reaction in the spinel reaches its equilibrium ($\partial \Delta G_{D}/\partial Q=0$), Equation (16) becomes:

(17) $$\frac{\partial \Delta G_{D}}{\partial Q}=0=-1+\frac{a}{h}\left(T-T_{c}\right)Q+\frac{c}{h}Q^{5}.$$

Substituting Q = 1 at 0 K into Equation (17) leads to *a*/h = (*c*/*h* − 1)/*T*_c_, and Equation (17) may be rewritten as [57]:

(18) $$T=T_{C}+\frac{T_{C}}{\left(c^{\prime }-1\right)Q}\left(1-c^{\prime }Q^{5}\right),$$

where $c^{\prime }=c/h$. 

Using a weighted least-squares regression method, we fit our *x*-*T* data to Equation (18) and obtain $T_{C}$ = 2.2(1825) K and $c^{\prime }$ = 1.00(65) (R^2^ = 0.937) (Figure 5b). In comparison, Redfern et al. [43] obtained $T_{C}$ = 445(109) K and $c^{\prime }$ = 1.62(21) for the sample S, and $T_{C}$ = -122(151) K and $c^{\prime }$ = 0.87(15) for the sample N. Within uncertainty, these parameters can be viewed as identical.

The Carpenter and Salje model [51] we have calibrated is compared in Figure 5b to the Navrotsky and Kleppa model [49] and the O’Neill and Navrotsky model [50] constrained above. These models are almost identical at *T* > ~1000 K. However, at *T* < ~800 K, the Carpenter and Salje model [51] shows significant difference with both the Navrotsky and Kleppa model [49] and the O’Neill and Navrotsky model [50]. On account of the lack of the *x*-*T* data at very low temperatures (*T* < 873 K), it is hard to judge whether the Carpenter and Salje model [51] is more appropriate or not. Besides, due to the still narrow range of our *x*-*T* data (0.18(1) < *x* < 0.357(60) and 873 < *T* < 1887 K) gathered from different data resources, the derived value of the $T_{C}$ has a large uncertainty. Accurate *x*-*T* data, especially those at very low *T*, are badly needed in order to better constrain the parameters of these thermodynamic models, so that these models will eventually be applicable in a wide temperature range.

## 3. Discussion

### 3.1. Evaluation for the Constituent Items of $\Delta G_{D}$ and the Contribution of $T\Delta S_{D}$

The constituent items of the $\Delta G_{D}$ (Equation (11)) were differently described according to their relationships with the *x*, with some of them sometimes neglected, which eventually resulted in different thermodynamic models. In this section, we evaluate these items in Equation (11) and discuss whether the $T\Delta S_{D}$ can be neglected or not using experimental data from different research fields.

Let us discuss the $P\Delta V_{D}$ term first. According to the correlation between the *a* and *x* established with some quenched samples by Andreozzi et al. ([29]; *a* = 8.0900 − 0.0242*x*), the unit-cell volume difference between the fully ordered structure (*x* = 0) and the fully disordered structure (*x* = 2/3) is approximately −3.16 Å^3^. The volume change ($\Delta V_{D}$) for the cation exchange reaction is therefore −2.38 × 10^−7^ m^3^·mol^−1^, and the corresponding energy change ($P\Delta V_{D}$) at room pressure is −0.024 J·mol^−1^ only. Further, the volume variation of the accompanying thermal expansion is 2.98 × 10^−6^ m^3^·mol^−1^ according to Carbonin et al. [24], and the corresponding energy change ($P\Delta V_{T}$) at room pressure is 0.298 J·mol^−1^. Compared to the magnitudes of the $\Delta U_{D}$ and the $T\Delta S_{C}$ (kJ·mol^−1^), the $P\Delta V_{D}$ term is indeed negligible and the approximation $\Delta H_{D}\approx \Delta U_{D}$ is reasonable. When the cation exchange reaches the equilibrium at certain temperature and pressure ($\partial \Delta G_{D}/\partial x=0$), we then have:

(19) $$\Delta G_{D}=\Delta H_{D}-T\left(\Delta S_{C}+\Delta S_{D}\right).$$

**Figure 7:**
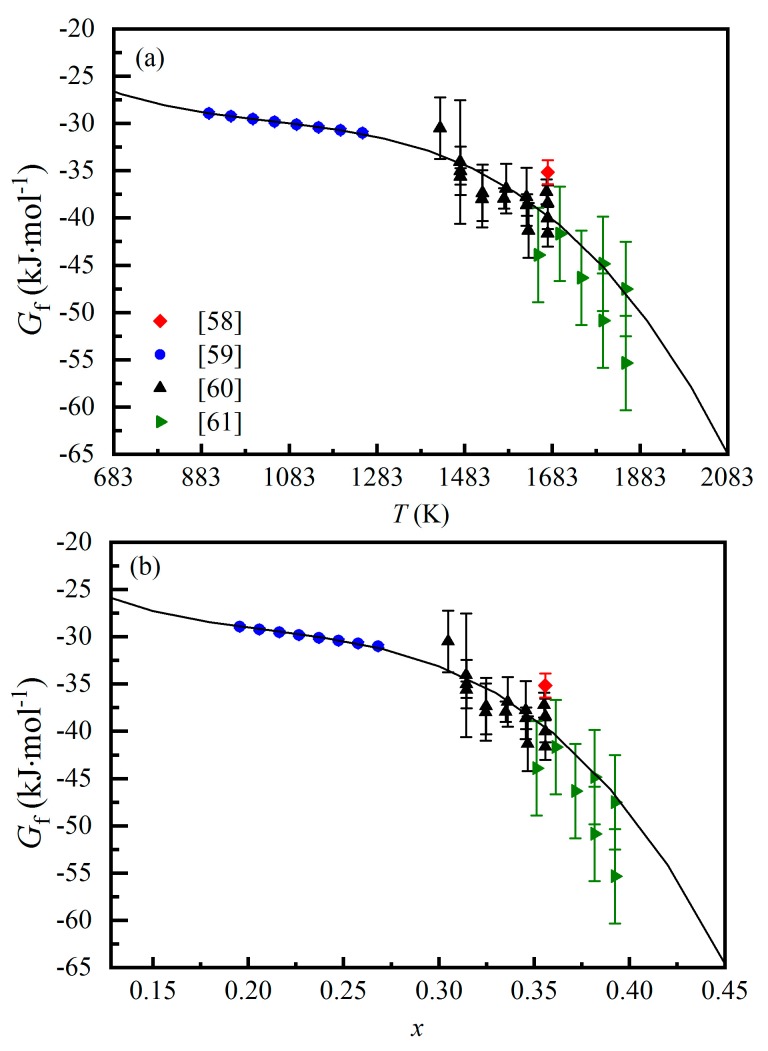
(**a**) Correlation between the $G_{f}$ and temperature (*T*) established with the experiment data from four different studies. (**b**) Correlation between the $G_{f}$ and *x*.

The $\Delta G_{D}$ caused by the cation exchange reaction in the MgAl_2_O_4_-spinel can be evaluated by resorting to the difference of the Gibbs energies of formation of the MgAl_2_O_4_-spinels ($\Delta G_{f}$) at different temperatures. Rosén and Muan [58], Chamberlin et al. [59], Jacob et al. [60], and Fujii et al. [61] determined the values of the $G_{f}$ at different temperatures using different methods, as summarized in Figure 7a. Fitting these experimental results leads to the following $G_{f}$~*T* correlation, $G_{f}$ = −2.613 × 10^−8^ × *T*^3^ + 8.159 × 10^−5^ × *T*^2^ − 0.090 × *T* + 5.281. In addition, our *x*-*T* data in the *T* range of ~873–1887 K (Figure 5b) can be well described by the equation *x* = 2.073 × 10^−4^ × *T* + 0.899 × 10^−3^. Therefore, we have a $G_{f}$-*x* correlation as $G_{f}$ = −2.134 × 10^3^ × *x*^3^ + 1.351 × 10^3^ × *x*^2^ − 310.53 × *x* − 3.88 (Figure 7b). Eventually the difference of the Gibbs energies of formation of the MgAl_2_O_4_-spinels ($\Delta G_{f}$) with different *x* at different temperatures can be calculated. For example, when the temperature of the MgAl_2_O_4_-spinel increases from ~900 K to ~1600 K, its corresponding *x* value changes from ~0.196 to ~0.340, and the $\Delta G_{f}$ is $G_{f-1600}$–$G_{f-900}$ = −8.38(662) kJ·mol^−1^. Since the Gibbs energy change caused by the temperature increase ($\Delta G_{T}$) has been found very small ($\Delta G_{T}$ = $P\Delta V_{T}$), the Gibbs energy change caused by the cation exchange reaction can be closely approximated as $\Delta G_{D}\approx \Delta G_{f}$.

Navrotsy and Kleppa [49] used the solution calorimetry to measure the enthalpies of solution of the MgAl_2_O_4_-spinels with different *x* values ($H_{S}$). Their studied materials were natural MgAl_2_O_4_-spinels with some compositional impurities such as Si, Fe, V, Cr, etc. We assume that these impurities would not significantly influence the cation exchange reaction of the MgAl_2_O_4_-spinels and the associated $\Delta H_{S}$. The starting *x* was unknown. Considering that prolonged geological processes might allow the cation exchange reaction of natural MgAl_2_O_4_-spinels to proceed towards very low *x* values (e.g., *x* = 0.125, Carbonin et al. [21]; *x* = 0.23, Lucchesi and Della Giusta [22]; *x* = 0.05, Maekawa et al. [23]; *x* = 0.130, Carbonin et al. [24]; *x* = 0.145, Nestola et al. [25]; *x* = 0.145, Liu et al. [26]), we have taken ~0.12 as the initial *x* value, which nevertheless does not influence the following discussion at all. The MgAl_2_O_4_-spinels were first heated at 1008, 1143, 1333, and 1573 K for 24 h. According to Figure 4c,d, the MgAl_2_O_4_-spinels annealed at 1143, 1333, and 1573 K should have reached their cation exchange equilibrium, and their *x* values should be, respectively, 0.246(10), 0.292(10), and 0.339(10), according to the O’Neill and Navrotsky model [50] calibrated in this study. Then, these annealed MgAl_2_O_4_-spinels were completely dissolved in a lead-cadmium-borate melt (of composition 9PbO·3CdO·4B_2_O_3_) at 968 K for 30~60 min [62], and their $H_{S}$ values were measured as 51.53(29), 49.81(33), and 50.15(29) kJ·mol^−1^, respectively. According to the kinetics results (Figure 4a), it is clear that this dissolving process would not have significantly changed the *x* value of the diminishing residual samples. On the other hand, Navrotsy and Kleppa [49] did not report any details about the quench process for their annealed samples, so that the final *x* values were somewhat uncertain. Indeed, the $H_{S}$ value of the MgAl_2_O_4_-spinel annealed at 1573 K, which was most likely affected by the quench process, was very close to that of the MgAl_2_O_4_-spinel annealed at 1333 K, nullifying the usefulness of this $H_{S}$ measurement. With the rest two $H_{S}$ measurements, $H_{S}$ = 51.51(29) kJ·mol^−1^ (*x* = 0.246(10)) and $H_{S}$ = 49.79(33) kJ·mol^−1^ (*x* = 0.292(10)), we can derive the $H_{S}$-*x* correlation, from which a $\Delta H_{D}$ of −29.04(1817) kJ·mol^−1^ has been calculated for the spinel changing its *x* value from 0 to 2/3. For the most interested temperature range of ~900–1600 K, a $\Delta H_{D}$ of −6.32(1462) kJ·mol^−1^ should be obtained as the *x* value changes from ~0.196 to ~0.340.

The $T\Delta S_{C}$ caused by the cation exchange reaction can be straightforwardly calculated using Equation (13). For example, it is 12.155 kJ·mol^−1^ as the *x* value of the MgAl_2_O_4_-spinel changes from ~0.196 to ~0.340 (*T* changing from ~900 to ~1600 K).

Rearranging Equation (11) leads to $T\Delta S_{D}=\Delta G_{D}-\Delta H_{D}+T\Delta S_{C}$. As the temperature increases from ~900 K to ~1600 K, the equilibrium *x* value of the MgAl_2_O_4_-spinel changes from ~0.196 to ~0.340, the $H_{D}$ value changes by −6.32(1462) kJ·mol^−1^, the $G_{D}$ value varies by −8.38(662) kJ·mol^−1^, the $T\Delta S_{C}$ value alters by 12.155 kJ·mol^−^^1^, and the $T\Delta S_{D}$ change is then ~10.095 kJ·mol^−1^. Clearly, the magnitude of the $T\Delta S_{D}$ is so large that the $T\Delta S_{D}$ term in Equation (19) should not be neglected.

As disclosed by the above case analysis, the $\Delta G_{f}$ value and the $\Delta H_{D}$ value have the same order of magnitudes, which requires the $\Delta S_{D}$ value and the $\Delta S_{C}$ value to attain the same order of magnitudes (Equation (19)). However, the $\Delta H_{D}$ value still bears large uncertainty. More and highly accurate measurements on this line are obviously welcomed, in order to make sure whether the $T\Delta S_{D}$ term in Equation (19) can be neglected or not.

If the $T\Delta S_{D}$ term cannot be neglected indeed, Equation (11) should then be expressed as:

(20) $$-RTln\left(\frac{x^{2}}{\left(1-x\right)\left(2-x\right)}\right)=\alpha +2\beta x-T\left(\frac{\partial \Delta S_{D}}{\partial x}\right).$$

Fitting our *x*-*T* data to this equation, we have obtained α = 24.59(33) kJ·mol^−1^, β = −45.42(158) kJ·mol^−1^, and $\Delta S_{D}$ = −23.48(71) J·mol^−1^·K^−1^. Rather interestingly, Maekawa et al. [23] reported similar results, α = 30.5 kJ·mol^−1^, β = −30 kJ·mol^−1^, and $\Delta S_{D}$ = −13 J·mol^−1^·K^−1^. It should be pointed out that our fitted value of the $\Delta S_{D}$ is very close to the value independently estimated using the experimental data of the reaction enstatite + spinel = pyrope + forsterite by Wood et al. ([47]; −25.12 J·mol^−1^·K^−1^). In addition, our non-negligible $\Delta S_{D}$ seems in general agreement with the experimental results obtained for the cation-disordering equilibrium in magnetite by Wu and Mason [63], according to the analysis carried out by O’Neill and Navrotsky ([50]; −13.39 J·mol^−1^·K^−1^).

Wood et al. [47] proposed that the $\Delta S_{D}$ might have two potential physical origins. One was the vibrational entropy change caused by the cation exchange reaction, and the other was potential short-range Mg-Al order in the spinel structure which affects the $\Delta S_{C}$. Wood et al. [47] and Maekawa et al. [23] argued for the latter, with their calculations based on the assumption that the short-range order did exist. However no independent evidence for the existence of any short-range order has been obtained so far [59,64]. On the other hand, Raman spectroscopic studies have shown that the Mg-Al cation exchange reaction practically causes some variations in the frequencies, intensities, widths, and asymmetries of the relevant Raman peaks, and even brings forth new Raman peaks [26,35,38]. The real origin of the $\Delta S_{D}$ is presently still at large.

No experimental measurements on the non-configurational entropy ($\Delta S_{D}$) have been performed. Bonnickson [65], King [66], Richet and Fiquet [67], and Klemme and Ahrens [68], measuring the entropies or the enthalpies of some MgAl_2_O_4_-spinels at different temperatures, would have provided some clues if the *x* values of the used materials were quantified. Unfortunately, only Klemme and Ahrens [68] characterized the starting *x* value of their MgAl_2_O_4_-spinel, which should have remained constant during the whole measuring process (*T* range of 4.33~305.2 K). All other studies did not report the starting *x* values. Furthermore, it is also impossible to estimate the starting *x* values due to the lack of detailed descriptions on how the starting MgAl_2_O_4_-spinels were prepared and quenched. More experiments with adequate characterization of the starting *x* values and full description of the experimental process should be very useful in regarding to constraining the $\Delta S_{D}$.

### 3.2. Pressure Dependence of the x

Compared to the knowledge accumulated for the effect of *T*, little is known about how pressure affects the Mg-Al cation exchange reaction of the MgAl_2_O_4_-spinel.

The only experimental study on the Mg-Al cation exchange reaction of the MgAl_2_O_4_-spinel at simultaneously high temperature and high pressure was performed in situ by Méducin et al. [46]. The characterizing method was the neutron powder diffraction, and the *P*-*T* conditions were 0–3.2 GPa and 0–1600 K, respectively. The experiments were conducted from low *T* to high *T* (with accompanying *P* increase) and then from high *T* to low *T* (with accompanying *P* decrease), with unknown experimental details such as the heating ramp, the waiting time before collecting the neutron diffraction data, and the data-collecting time at high *P*. If we assume that the experiment conditions in Méducin et al. [46] were similar to those in Redfern et al. [43] (e.g., a data-collecting time of ~40 min), and that the reaction rates at high *P* were not so different to those at ambient *P* (Figure 4), we could have reached the conclusion that, with the exceptions of three experiments at 571(5) K and 0.4(1) GPa, 789(7) K and 0.4(1) GPa, and 390(4) K and 0.5(1) GPa, all other experiments should have reached the Mg-Al cation exchange equilibrium. Interestingly, the experiments did not show obvious changes of the *u* parameters, which were explained as a counterbalanced result of structural changes caused by cation exchange, volume compression due to increasing pressure, and thermal expansion. Méducin et al. [46] argued that high *P* significantly enhanced the Mg-Al disordering process (i.e., *x* becoming much larger at high *P* than at ambient *P* for similar *T*). Fitting their experiment data within the 789–1591 K interval to the O’Neill and Navrotsky model [50], Méducin et al. [46] obtained α = 31(6) kJ·mol^−1^ and β = −20(13) kJ·mol^−1^.

**Figure 8:**
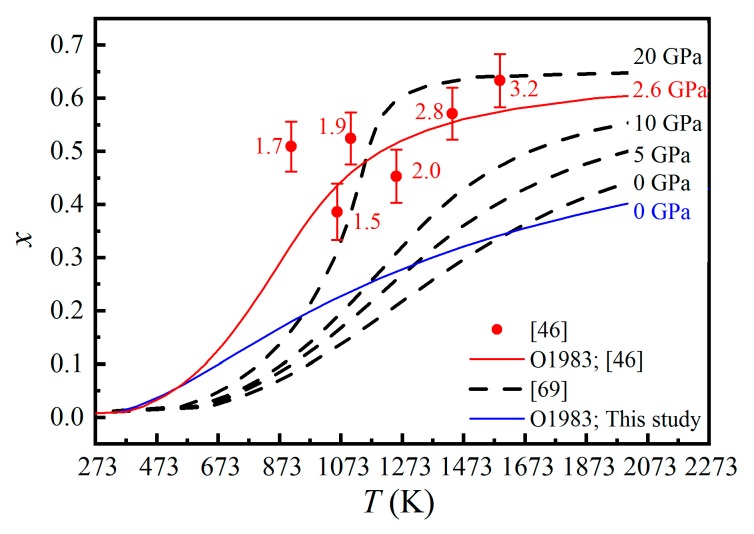
Correlations between temperatures (*T*), pressure (*P*) and *x* of the MgAl_2_O_4_-spinel. Red points stand for the equilibrium experiment data for the Mg-Al cation exchange reaction acquired by Méducin et al. [46], which passed our evaluation. Numbers beside them are pressures in GPa for the experiments. Red solid line refers to the O’Neill and Navrotsky thermodynamic model [50] calibrated for a pressure of 2.6 GPa by Méducin et al. [46]. Black dashed lines stand for the thermodynamic models at different *P* theoretically obtained by Da Rocha and Thibaudeau [69]. Blue line refers to the O’Neill and Navrotsky thermodynamic model [50] calibrated by our *x*-*T* data.

Da Rocha and Thibaudeau [69] theoretically studied the cation exchange reaction of the MgAl_2_O_4_-spinel at high pressure. Their results suggested that the $P\Delta V_{D}$ term should not be neglected under high pressure, and consequently modified the O’Neill and Navrotsky model [50] (Equations (11) and (15)) by introducing $\Delta V=\mu x+\nu x^{2}$. The α and β parameters were accordingly modified as $\alpha^{\prime }=\alpha +P\mu$, $\beta^{\prime }=\beta +P\nu$. In this way, the correlation between the temperature, pressure and *x* could be probed. By performing *ab initio* calculations, Da Rocha and Thibaudeau [69] calculated the values of the $\alpha^{\prime }$ and $\beta^{\prime }$ parameters at 0, 5, 10 and 20 GPa. However, their simulating results are very different to the experimental results of Méducin et al. [46], as shown in Figure 8.

In conclusion, the effect of pressure on the Mg-Al cation exchange reaction is presently unclear. More studies at simultaneously high *P* and high *T* should be carried out before accurate thermodynamic model at high *P* can be constructed.

### 3.3. Composition Influence on the x

The MgAl_2_O_4_-spinels may contain some chemical impurities and structural vacancies, and thus show certain compositional deviation from the standard stoichiometry. It is well known that there is a series of solid solutions along the MgAl_2_O_4_-□_1/3_Al_8/3_O_4_ join (□ stands for the structural vacancy), with the unit-cell parameter *a* varying from ~8.084 Å towards ~7.908 Å [64,70,71]. As suggested by Peterson et al. [42], O’Neill [72], Redfern et al. [43] and Andreozzi et al. [29], some structural vacancies in the MgAl_2_O_4_-spinel might significantly influence the Mg-Al cation exchange reaction, which, however, needs to be verified by further systematic experimental investigations.

A distinct chemical impurity in the natural MgAl_2_O_4_-spinels is trace Cr^3+^. Due to its much lower octahedral site preference energy and larger ionic radius compared to the Al^3+^ [49,50], the Cr^3+^ occupies the M-sites only and enlarges the *a* value of the MgAl_2_O_4_-spinels (*a* = 0.124 × Cr(apfu) + 8.087; Bosi and Andreozzi [73]). According to Princivalle et al. [74], small amounts of Cr^3+^ would significantly increase the Mg-Al cation exchange reaction rates *K*_1_ and *K*_2_, and decrease the equilibrium *x* value of the MgAl_2_O_4_-spinel at certain *T*.

**Figure 9:**
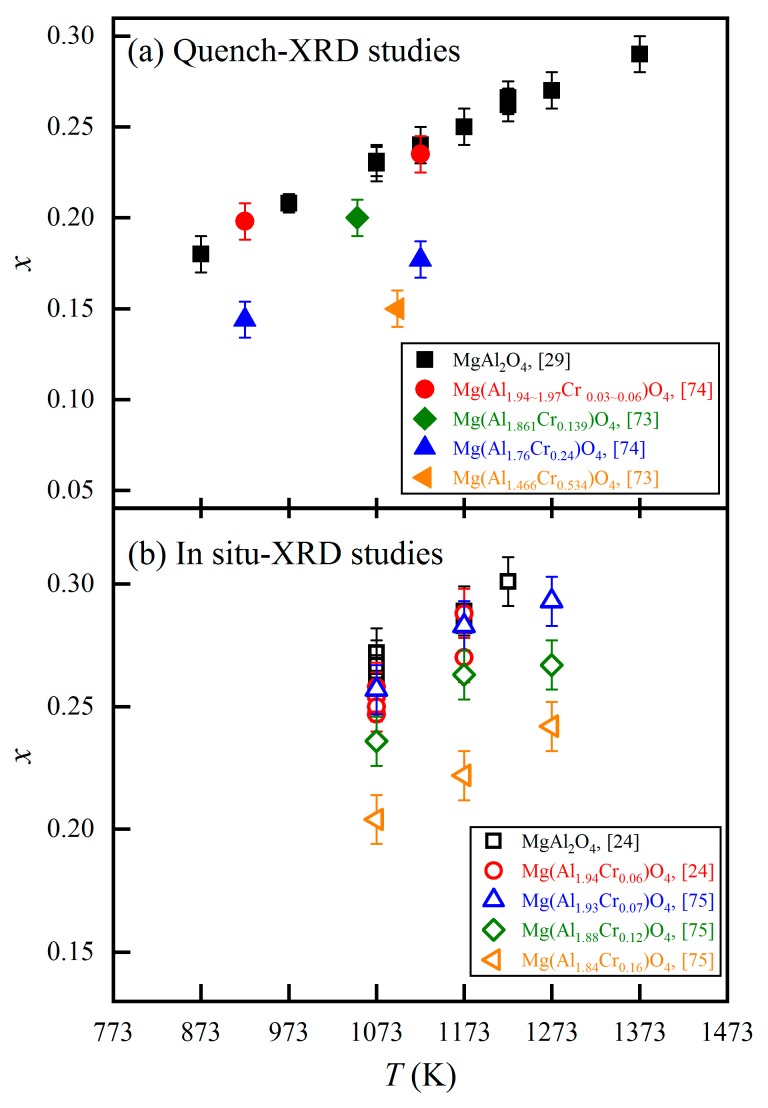
Correlations between temperatures (*T*) and *x* values of the MgAl_2_O_4_-rich spinels containing different amounts of Cr^3+^. (**a**) Data from quenched samples; (**b**) data from in situ measurements. The *x* values shown were all obtained by single-crystal X-ray diffraction.

We have collected some *x~T* data of the Mg-Al cation exchange reaction in the MgAl_2_O_4_-rich spinels containing different contents of Cr^3+^ from related studies [24,29,73,74,75]. Similar screening criteria as those used in the pure MgAl_2_O_4_-spinel case were applied to them (i.e., cation exchange equilibrium, quenching modification, accuracy in temperature measurement). The final *x*-*T* data, both obtained from quenched samples and in situ acquired at high *T*, are summarized, respectively, in Figure 9a,b. According to Figure 9, small amounts of Cr^3+^ significantly influence the *x* values at certain temperatures indeed. It follows that further systematic investigations on the Mg-Al cation exchange equilibrium in the MgAl_2_O_4_-rich spinels with different amounts of Cr^3+^ are desirable.

### 3.4. Some Implications of the Cation Order-Disorder Reaction in Spinel

Spinel has many useful chemical and physical properties and is widely employed in various industries. In geology, the spinel is very useful as a petrogenesis indicator to provide genetic information about the chemical composition, oxygen fugacity, crystallizing temperature, cooling rate of the parent magmas [76,77,78,79,80,81,82], and to help exploring interactions between peridotite xenoliths and entraining magmas [79,83,84], etc.

Many spinel-bearing phase assemblages have been calibrated as geothermometers, geobarometers, and oxygen barometers [13,14,15,16,80,85,86]. Recently Della Giusta et al. [87] and Princivalle et al. [88] put forward a geothermometer solely based on the correlation between the Mg-Al cation exchange reaction of the spinel and temperature:

(21) T=6640[ATlAltot+0.101(1−MTg−ATl)+0.041(2−AMl−MMg)].

The material used to calibrate this thermometer was an Mg-Al rich spinel with small amounts of Fe^2+^, Fe^3+^, Cr^3+^, and Ni^2+^, etc. The claimed accuracy of this geothermometer was ±20 °C. It should be noted that this type of thermometer constrains the closure temperature instead of the crystallization temperature of the spinel. According to the kinetics studies, the closure temperature depends on the cooling rate. Slow and prolonged cooling processes would provide more time for the cation exchange reaction of the spinel to proceed to much low extents, and the closure temperature estimated by the thermometer would be low, and vice versa. Thus this thermometer is most useful in exploring the cooling processes of the relevant geological bodies.

Spinel can incorporate a large number of trace elements in its structure, and the partitioning behavior of these trace elements between the spinel and magmas can provide important constraints on the evolution process of the magmas [89,90,91]. According to previous studies [92,93], the partitioning behavior of trace elements between the spinel and magmas is mainly influenced by temperature, pressure, oxygen fugacity, the composition of spinel, and so on. Here, we suggest that the cation exchange reaction in the spinel would also influence the partitioning behavior. The partitioning of a trace element between a mineral and melt can be expressed as [94]:

(22) $$D_{i}=D_{0}\exp\left(\frac{-4\pi EN_{A}\left(\frac{r_{0}}{2}{\left(r_{i}-r_{0}\right)}^{2}+\frac{1}{3}{\left(r_{i}-r_{0}\right)}^{3}\right)}{RT}\right),$$

where $N_{A}$ is the Avogadro’s number, Ε is the Young’s Modulus of the crystallographic site, R is the gas constant, and *T* is temperature in K. $D_{i}$ and $D_{0}$ are the partition coefficients of the substituting cation *i* (with the radius of $r_{i}$) and of the substituted cation on the specific crystallographic site (with the optimum radius of $r_{0}$), respectively. Clearly, the partitioning behaviors of the trace elements can be strongly affected by the sizes and elastic properties of the sites incorporating them. In the case of the spinel, any variation of the *x* value induced by varying *T* and *P* will alter the sizes of the T-sites and the M-sites [29,43], and then will change the partition coefficients of the trace elements. Due to the commonly small grain sizes of the spinels crystallizing from the experimental melts [95], however, the variations of the partition coefficients with *T* and *P* have not been well studied.

## 4. Conclusions

The MgAl_2_O_4_-spinel is widely applied in various industries due to its novel physical and thermal properties. However, these properties can be influenced by the crystal structure change due to the inter-site Mg-Al cation exchange. The Mg-Al cation exchange reaction is a complex function of temperature, pressure and reaction time. With detailed calculation and comparison, this review has shown that the results of Andreozzi and Princivalle [45] better reflect the evolution of the *x* with time at the investigated *T*. The kinetics results show that the Mg-Al cation exchange reaction in the MgAl_2_O_4_-spinel proceeds extremely fast at high temperatures and extremely slow at low temperatures. In view of such a reacting feature, we evaluated all previous equilibrium experiments, and focused mainly on two aspects: (1) Whether the cation exchange reaction closely approached its equilibrium within the heating duration, and (2) whether the quench process fully preserved the cation distribution state attained at high temperature. Besides, we also carried out necessary temperature correction and data elimination. These practices have finally led to a reliable *x*-*T* dataset (71 data pairs in total). Fitting these data to the thermodynamic model equations, we have obtained $\Delta H_{D,\mathrm{int}}$ = 29.30(19) kJ·mol^−1^ for the Navrotsky and Kleppa model [49], α = 28.63(136) kJ·mol^−1^ and β = 1.35(261) kJ·mol^−1^ for the O’Neill and Navrotsky model [50], and $T_{C}$ = 2.2(1825) K and $c^{\prime }$ = 1.00(65) for the Carpentar and Salje model [51]. These three models are almost identical at *T* > ~1000 K. However, at *T* < ~800 K, the Carpentar and Salje model [51] shows significant difference to the others, which needs to be verified with more experimental data, especially with those at relatively low *T*. In addition, we evaluated the constituent items of the Gibbs free energy caused by the Mg-Al cation exchange reaction of the MgAl_2_O_4_-spinel with the experimental results from different research fields. We have found that the $\Delta G_{D}$ value and the $\Delta H_{D}$ value caused by the Mg-Al cation exchange reaction are of the same order of magnitudes, and, therefore, the $\Delta S_{D}$ value and the $\Delta S_{C}$ value should be similar. In this situation, the $\Delta S_{D}$ item should not be neglected. Future systematic theoretical studies may help resolve the $\Delta S_{D}$ puzzle. Our review also shows that the studies under high pressure are of great shortage, with their results in apparent discrepancy. More experimental data at high *P* are strongly needed in order to ascertain the Mg-Al cation exchange reaction in the MgAl_2_O_4_-spinel under various *P*/*T* conditions, especially those prevailing within the deep Earth.

## Supplementary Materials

The following is available online, Table S1: Some thermodynamic equilibrium experiments details and results.
