# Supplementary material for: Kinetics and Thermodynamics of Mg-Al Disorder in MgAl2O4-Spinel: A Review
Source: Molecules. 2019 May 1;24(9):1704. doi: 10.3390/molecules24091704 (PMC6540108; doi:10.3390/molecules24091704)
Supplement: Supplementary file 1 [file molecules-24-01704-s001.pdf]

Supplementary Material for

Kinetics and Thermodynamics of Mg-Al Disorder in $MgAl_2O_4$-Spinel: A Review

Yunlu Ma [1,2] and Xi Liu [1,2,*]

[1] School of Earth and Space Sciences, Peking University, Beijing 100871, China; Yunlu.Ma@pku.edu.cn (Y.M.); Xi.Liu@pku.edu.cn (X.L.)
[2] Key Laboratory of Orogenic Belts and Crustal Evolution, Ministry of Education of China, Beijing 100871, China

* Correspondence: Xi.Liu@pku.edu.cn; Tel.: +86-10-6275-3585; Fax: +86-10-6275-2996

Supplementary Table 1

Some experimental details and results of the 1 atm thermodynamic equilibrium experiments for the Mg-Al cation exchange reaction in the $MgAl_2O_4$-spinel, which pass our evaluation (84 experiments in total).

D/O[a]	*T* (°C)	*T* (K)	Time	x_i[b]	x_f[c]	*T*-corrected (K)[d]
**Wood et al. [1]: ^{27}Al MAS-NMR; Sample quenched within ~2 s**						
D	715(10)[e]	988(10)	370 h	0.21(2)	0.24(2)	
D	800(10)	1073(10)	210 h	0.21(2)	0.32(3)	
D	850(10)	1123(10)	70 h	0.21(2)	0.35(4)	
D	900(10)	1173(10)	185 h	0.21(2)	0.39(4)	
D	1050(10)	1323(10)	400 h	0.21(2)	0.39(4)	
O	715(10)	988(10)	370 h	0.39(4)	0.26(3)	
O	800(10)	1073(10)	3 h	0.37(4)	0.32(3)	
O	800(10)	1073(10)	210 h	0.37(4)	0.30(3)	
O	850(10)	1123(10)	70 h	0.36(4)	0.35(4)	
O	1050(10)	1323(10)	400 h	0.37(4)	0.36(4)	
O	1200(10)	1473(10)	210 h	0.39(4)	0.37(4)	
**Peterson et al. [2]: Neutron powder diffraction; In situ measurement**						
O	900	1173	~40 min	0.36(1)	0.33(2)	
O	1000	1273	~40 min	0.32(2)	0.36(1)	
**Millard et al. [3]: ^{27}Al and ^{17}O MAS-NMR; Sample quenched within ~5-10 s for experiments at $T < 950$ K, and ~2 s for $T > 950$ K**						
O	700(5)	973(5)	409 h	0.26(3)	0.22(3)	
O	800(5)	1073(5)	498 h	0.26(3)	0.26(3)	
O	807(5)	1080(5)	180 h	0.26(3)	0.25(3)	
O	902(5)	1175(5)	471 h	0.26(3)	0.25(3)	
O	902(5)	1175(5)	528 h	0.30(3)	0.27(3)	
D	1002(5)	1275(5)	166 h	0.26(3)	0.29(3)	
D	1004(5)	1277(5)	186 h	0.26(3)	0.29(3)	

D	1201(5)	1474(5)	138 h	0.26(3)	0.28(3)	
D	1202(5)	1475(5)	71-87 h	0.26(3)	0.29(3)	
D	1202(5)	1475(5)	220 h	0.26(3)	0.30(3)	

Maekawa et al. [4]: ^{27}Al MAS-NMR; In situ measurement

	1211(12)	1484(15)			0.307(40)	
	1240(12)	1513(15)			0.296(40)	
	1301(13)	1574(16)			0.331(40)	
	1380(14)	1653(17)			0.313(40)	
	1410(14)	1683(18)			0.338(60)	
	1430(14)	1703(17)			0.314(60)	
	1430(14)	1703(17)			0.357(60)	
	1517(15)	1790(18)			0.341(60)	
	1614(16)	1887(19)			0.327(60)	

Redfern et al. [5]: Neutron powder diffraction; In situ measurement

Sample S

D	1018	1291	~40 min	0.206(11)	0.234(11)	1114(50)
D	1082	1355	~40 min	0.234(11)	0.236(13)	1167(50)
D	1145	1418	~40 min	0.236(13)	0.249(12)	1219(50)
D	1208	1481	~40 min	0.249(12)	0.271(12)	1271(50)
D	1271	1544	~40 min	0.271(12)	0.268(12)	1322(50)
D	1333	1606	~40 min	0.268(12)	0.286(12)	1373(50)
D	1371	1644	~40 min	0.286(12)	0.294(12)	1405(50)
D	1389	1662	~40 min	0.294(12)	0.298(12)	1419(50)
O	1322	1595	~40 min	0.296(12)	0.275(12)	1364(50)
O	1259	1532	~40 min	0.275(12)	0.277(12)	1313(50)
O	1195	1468	~40 min	0.277(12)	0.279(12)	1260(50)
O	1132	1405	~40 min	0.279(12)	0.245(16)	1208(50)
O	1005	1278	~40 min	0.245(16)	0.214(24)	1104(50)
O	941	1214	~40 min	0.214(24)	0.207(11)	1051(50)
O	879	1152	~40 min	0.207(11)	0.202(11)	1000(50)
D	1000	1273	~40 min	0.194(11)	0.205(11)	1100(50)
D	1100	1373	~40 min	0.205(11)	0.230(11)	1182(50)
D	1200	1473	~40 min	0.230(11)	0.236(11)	1264(50)
D	1300	1573	~40 min	0.236(11)	0.255(13)	1346(50)
D	1400	1673	~40 min	0.255(13)	0.298(11)	1429(50)
D	1500	1773	~40 min	0.298(11)	0.336(11)	1511(50)
D	1600	1873	~40 min	0.336(11)	0.347(12)	1593(50)

Sample N

D	1038	1311	~40 min	0.242(12)	0.286(12)	1139(50)
D	1103	1376	~40 min	0.286(12)	0.269(12)	1184(50)
D	1168	1441	~40 min	0.269(12)	0.283(12)	1238(50)
D	1233	1506	~40 min	0.283(12)	0.287(12)	1291(50)
D	1300	1573	~40 min	0.287(12)	0.301(12)	1346(50)
D	1366	1639	~40 min	0.301(12)	0.320(14)	1401(50)

D	1403	1676	~40 min	0.320(14)	0.288(13)	1431(50)
D	1398	1671	~40 min	0.288(13)	0.323(13)	1427(50)
O	1349	1622	~40 min	0.323(13)	0.293(13)	1387(50)
O	1284	1557	~40 min	0.293(13)	0.300(12)	1333(50)
O	1219	1492	~40 min	0.300(12)	0.302(12)	1280(50)
O	1154	1427	~40 min	0.302(12)	0.288(12)	1226(50)
O	1089	1362	~40 min	0.288(12)	0.280(12)	1173(50)
O	1026	1299	~40 min	0.280(12)	0.262(12)	1121(50)
**Andreozzi et al. [6]: Single-crystal X-ray diffraction; Sample quenched within < 0.5 s**						
D	800(10)	1073(10)	1 d	0.229(6)	0.231(8)	
D	850(10)	1123(10)	1 d	0.229(6)	0.24(1)	
D	900(10)	1173(10)	1 d	0.229(6)	0.25(1)	
D	950(10)	1223(10)	1 d	0.229(6)	0.262(9)	
D	1000(10)	1273(10)	1 d	0.229(6)	0.27(1)	
D	1100(10)	1373(10)	1 d	0.229(6)	0.29(1)	
O	950(10)	1223(10)	3 d	0.29(1)	0.266(9)	
O	800(10)	1073(10)	7 d	0.29(1)	0.23(1)	
O	700(5)	973(5)	90 d	0.29(1)	0.208(5)	
O	600(5)	873(5)	45 d	0.29(1)	0.18(1)	
**Carbonin et al. [7]: Single-crystal X-ray diffraction; In situ measurement**						
D	800(10)	1073(10)	2 h	0.229	0.261	1123(50)
D	900(10)	1173(10)	2 h	0.261	0.283	1223(50)
D	950(10)	1223(10)	2 h	0.283	0.301	1273(50)
O	900(10)	1173(10)	2 h	0.301	0.289	1223(50)
O	800(10)	1073(10)	2.2 h	0.289	0.272	1123(50)
O	800(10)	1073(10)	4.2 h	0.272	0.267	1123(50)

[a] D/O standing for cation disordering/ordering experiment.

[b] x_i representing the x value of the staring material.

[c] x_f representing the final x value of the experimental product.

[d] $T_{\text{-corrected}}$ representing T after some correction (see text for more discussion).

[e] Number in the parentheses representing one standard deviation to the rightmost digit.

References

1. Wood, B.J.; Kirkpatrick, R.J.; Montez, B. Order-disorder phenomena in $MgAl_2O_4$ spinel. *Am. Mineral.* **1986**, *71*, 999-1006.
2. Peterson, R.C.; Lager, G.A.; Hitterman, R.L. A time-of-flight neutron powder diffraction study of $MgAl_2O_4$ at temperatures up to 1273 K. *Am. Mineral.* **1991**, *76*, 1455-1458.
3. Millard, R.L.; Peterson, R.C.; Hunter, B.K. Temperature dependence of cation disorder in $MgAl_2O_4$ spinel using ^{27}Al and ^{17}O magic-angle spinning NMR. *Am. Mineral.* **1992**, *77*, 44-52.
4. Maekawa, H.; Kato, S.; Kawamura, K.; Yokokawa, T. Cation mixing in natural $MgAl_2O_4$ spinel: A high-temperature ^{27}Al NMR study. *Am. Mineral.* **1997**, *82*, 1125-1132, DOI: 10.2138/am-1997-11-1210.
5. Redfern, S.A.T.; Harrison, R.J.; O'Neill, H.St.C.; Wood, D.R.R. Thermodynamics and kinetics of cation ordering in $MgAl_2O_4$ spinel up to 1600 °C from in situ neutron diffraction. *Am. Mineral.* **1999**, *84*, 299-310, DOI: 10.2138/am-1999-0313.

6. Andreozzi, G.B.; Princivalle, F.; Skogby, H.; Della Giusta, A. Cation ordering and structural variations with temperature in $MgAl_2O_4$ spinel: An X-ray single-crystal study. *Am. Mineral.* **2000**, *85*, 1164-1171, DOI: 10.2138/am-2000-8-907.
7. Carbonin, S.; Martignago, F.; Menegazzo, G.; Dal Negro, A. X-ray single-crystal study of spinels: In situ heating. *Phys. Chem. Miner.* **2002**, *29*, 503-514, DOI: 10.1007/s00269-002-0262-6.
